# Comparative genomics of Alexander Fleming’s original *Penicillium* isolate (IMI 15378) reveals sequence divergence of penicillin synthesis genes

**DOI:** 10.1038/s41598-020-72584-5

**Published:** 2020-09-24

**Authors:** Ayush Pathak, Reuben W. Nowell, Christopher G. Wilson, Matthew J. Ryan, Timothy G. Barraclough

**Affiliations:** 1 Department of Life Sciences, Imperial College London, Silwood Park Campus, Ascot, Berkshire, SL5 7PY UK; 2 Department of Zoology, University of Oxford, 11a Mansfield Rd, Oxford, OX1 3SZ UK; 3 CABI, Bakeham Lane, Egham, Surrey, TW20 9TY UK

**Keywords:** Evolutionary genetics, Genome, Antimicrobials

## Abstract

Antibiotics were derived originally from wild organisms and therefore understanding how these compounds evolve among different lineages might help with the design of new antimicrobial drugs. We report the draft genome sequence of Alexander Fleming’s original fungal isolate behind the discovery of penicillin, now classified as *Penicillium rubens* Biourge (1923) (IMI 15378). We compare the structure of the genome and genes involved in penicillin synthesis with those in two ‘high producing’ industrial strains of *P. rubens* and the closely related species *P. nalgiovense*. The main effector genes for producing penicillin G (*pcbAB*, *pcbC* and *penDE*) show amino acid divergence between the Fleming strain and both industrial strains, whereas a suite of regulatory genes are conserved. Homologs of penicillin N effector genes *cefD1* and *cefD2* were also found and the latter displayed amino acid divergence between the Fleming strain and industrial strains. The draft assemblies contain several partial duplications of penicillin-pathway genes in all three *P. rubens* strains, to differing degrees, which we hypothesise might be involved in regulation of the pathway. The two industrial strains are identical in sequence across all effector and regulatory genes but differ in duplication of the *pcbAB*–*pcbC*–*penDE* complex and partial duplication of fragments of regulatory genes. We conclude that evolution in the wild encompassed both sequence changes of the effector genes and gene duplication, whereas human-mediated changes through mutagenesis and artificial selection led to duplication of the penicillin pathway genes.

## Introduction

Clinical use of antibiotics has revolutionised treatment of bacterial infection. The characterization of penicillin^1^ followed from Alexander Fleming’s discovery of lysis and inhibition of the growth of *Staphylococcus* on petri dishes colonized by the fungus *Penicillium rubens*^2^ (named at the time as *P. notatum,* and until recently as *P. chrysogenum*^3,4^). There followed a golden age in the use of antibiotics to combat bacterial disease^5^. It was soon realized, however, that this might be short-lived, as more and more pathogenic bacteria evolved resistance to these compounds^6–8^. The result is an arms race between clinical use of antibiotics and the evolution of resistance in target bacteria, which requires new classes of antibiotics and new methods of delivery if medicine is to retain the upper hand.

One potentially useful way to improve antibiotic use and deployment is to draw inspiration from the evolution of antibiotic production and resistance in nature. Micro-organisms produce antibiotics to suppress the growth of their antagonists and reduce competition for space and resources^9,10^. Accordingly, organisms are under selection to evolve resistance to the antibiotics of others, yet producers are under reciprocal selection to increase the efficacy of their own antibiotics against their antagonists. With such an arms race^11^, it is expected that as antibiotic resistance evolves, antibiotics themselves will evolve too^12^. Genome sequencing can reveal variation in antibiotic production pathways among organisms with different antibiotic profiles. For instance, the ability to produce new compounds might evolve by modification of the synthesis genes or metabolic pathways, or there might be changes to existing compounds to counter the degradation of the antibiotic by resistant bacteria. These adaptations could encompass changes in the amino acid sequence of effector genes, changes in regulators of the antibiotic-production pathway, or duplication of effector genes. Gene duplication might lead to increased expression of the enzyme if the copies are conserved, or to production of multiple variants of the antibiotic if paralogous copies diverge in function^13^. In recent decades, considerable effort has gone into the study of the evolution of antibiotic resistance as a solution to the crisis^7,14^, but the evolution of antibiotics themselves remains largely neglected. Understanding how antibiotics coevolve in natural arms races with resistant bacteria might help to design methods for countering resistance evolution in the parallel arms race in clinical settings.

**Figure 1:**
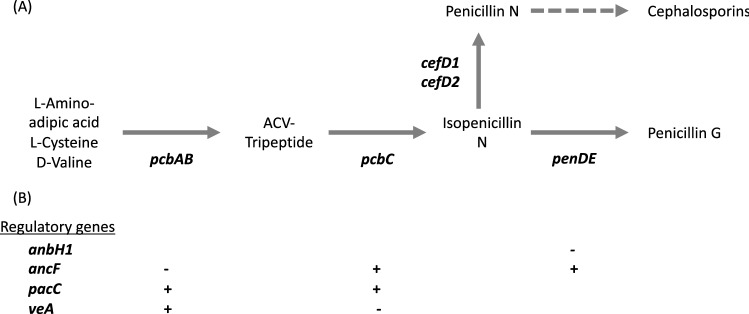
(**A**) Synthesis pathway for penicillin G and penicillin N. Bold italics indicate genes producing enzymes that catalyse each step (next to arrows). (**B**) Regulatory genes considered in this study that either upregulate (+) or downregulate (−) expression of the effector enzymes above.

In order to gain insights into the evolution of genes underlying the production of the classic antibiotic, penicillin, we here present a draft genome sequence for Fleming’s original isolate, *Penicillium rubens* (IMI 15378). Cryopreserved living samples of this isolate are kept in numerous global collections, and we revived the fungus from the CABI (IMI) living culture collection for DNA extraction and whole-genome sequencing. We compare the overall genome structure and variation in a set of genes involved in the penicillin-production pathway with closely related *Penicillium* isolates with sequenced genomes. In particular, we compare Fleming’s isolate to two industrial strains of *P. rubens* derived from a second isolation from the wild in the USA. These strains were originally misnamed as *P. chrysogenum* (and still are in some public sequence databases) but were subsequently shown by multigene analysis to belong to the *P. rubens* clade^3,4^. The original wild US isolate (NRRL1951), isolated from a mouldy cantaloupe, was subjected to multiple rounds of X-ray, chemical (chlormethine) and ultraviolet mutagenesis and artificial selection^15^ in order to generate isolates with high production rates of penicillin for industry^16^, including P2niaD18 and Wisconsin 54-1255^17^. The two lines split after an initial shared phase of both UV and X-ray mutagenesis and selection (via a common ancestor strain of Wis Q-176, see Fig. 1 in^16,18,19^). Wisconsin 54-1255 was derived from further rounds of UV and nitrogen mustard mutagenesis and selection, while P2niaD18 was derived from a separate round of undocumented improvements by Panlabs Inc, followed by deletion of the nitrate reductase gene niaD^16,18,19^. Any differences from the Fleming isolate that are shared by these two strains resulted either from evolution in the wild progenitors, or from the first steps of artificial selection and mutagenesis in the lab. In contrast, differences between the two industrial strains are solely the result of mutagenesis and artificial selection for high production: for example, comparison of P2niaD18 to the original Wisconsin 54-1255 genome^17^ revealed evidence for structural rearrangements and tandem duplication of penicillin-producing genes caused by the mutagenesis^20^. To provide a broader context for evolution in the wild, we also compare the Fleming genome to another penicillin-producing species from the *Penicillium* section *Chrysogena*, *P. nalgiovense*^21,22^, Our focus is primarily on short-term evolution among the closely related strains rather than the longer-term acquisition of penicillin production and so we do not repeat previous comparisons among more distantly related organisms^23,24^.

As well as comparing genome structure, we searched for genes involved in the penicillin pathway and compared copy number and sequence divergence among strains. Penicillin is a beta-lactam and encompasses several natural variations of the compound. *P. rubens* produces penicillin G via a 15 kb gene cluster of 3 genes (*pcbAB*, *pcbC* and *penDE*) present in the genomes of various filamentous fungi^25–27^ (Fig. 1). The first two genes *pcbAB* and *pcbC* encode the enzymes delta-(l-alpha-aminoadipyl)-l-cysteinyl-d-valine synthetase (ACVS) and isopenicillin N synthase respectively^23,28^, which catalyse the formation of the first bioactive molecule in the pathway, isopenicillin N^26^. All beta-lactams share this two-step pathway (including cephalosporins and cephamycins^23,26,28^). Evidence indicates that the genes *pcbAB* and *pcbC* were horizontally acquired from bacteria by the beta-lactam producing fungi^24^. The third gene *penDE* encodes the enzyme isopenicillin N acyltransferase, which catalyses the final step of the biosynthetic pathway that synthesizes penicillin G^27,29^. This gene is hypothesized to have evolved within the beta-lactam producing fungi rather than being horizontally acquired from bacteria^24^. In the beta-lactam producing fungi *Acremonium chrysogenum*, the genes *cefD1* and *cefD2* encode the isopenicillin N epimerase system and provide an alternative biosynthetic pathway to produce penicillin N from isopenicillin N, and cephalosporin C from penicillin N^30–32^. Several genes have been identified that play a role in regulating the pathway leading to penicillin G production, and evolved within beta-lactam producing fungi, described in more detail below^24,33^. We hypothesized that selection on antibiotic production should most likely result in changes in the coding sequence or copy number of effector genes at later stages of the pathway (i.e.* penDE*, which is unique to the production of penicillin G as opposed to other beta-lactams), or of the regulatory genes, rather than the upstream effector genes that generate pre-cursors used by multiple antibiotics.

## Materials and methods

### Culturing, DNA extraction and sequencing

The fungus *Penicillium rubens* Biourge (1923)^34^ IMI 15378 (= ATCC 8537; NRRL 824; CBS 205.57) was obtained from the CABI IMI culture collection. As part of a separate experiment (not reported here), replicates of fungus were grown for 11 weeks at 20 °C on petri dishes with LB Lennox agar media with addition of 20 g/L of sucrose. Fungus from each of 6 treatments was then cultured in LB Lennox Broth with 20 g/L of sucrose at room temperature for a week prior to the DNA extraction. For each of the six treatments, around 100 mg of washed mycelium was ground under liquid nitrogen and DNA was extracted using the DNeasy Plant Mini Kit (Qiagen). DNA libraries were prepared with an Illumina TruSeq PCR-free kit at the Department of Biochemistry, University of Cambridge, and sequenced with Illumina MiSeq v2 technology with 2 × 150 paired-end sequencing and 350 bp insert size. Separate library preparations and sequencing were performed for the 6 separate extractions, but subsequent results indicated no changes had accrued among the treatments, so reads were pooled for the assembly and analyses presented here.

### Genome assembly and analysis

Raw reads were filtered for low-quality bases and adapter sequences using BBTools ‘bbduk’ v38.22 (available at https://sourceforge.net/projects/bbmap/) with the parameters ‘ktrim = r k = 23 mink = 11 hdist = 2 maq = 10 minlen = 100 tpe tbo’. An initial assembly was constructed using SPAdes v3.13.0^35^ with default parameters and potential contamination was assessed using BlobTools v1.0^36^. Genome completeness was assessed using Benchmarking Universal Single-Copy Ortholog (BUSCO) gene sets v3.0.2 for Eukaryota (*n* = 303) and Fungi (*n* = 290)^37^. Assembled genomes for the two industrial strains, P2niaD18 and Wisconsin 54-1255, and *P. nalgiovense* (IBT 13039) were downloaded from NCBI GenBank (Table 1). The P2niaD18 genome was scaffolded into whole chromosomes in the source paper by comparing alignments to the Wisconsin 54-1255 genome. Genomes were aligned using nucmer in the mummer package 4.0beta, and structural changes visualized with dotplots using the DNANexus Dot browser (available at https://dnanexus.github.io/dot/). All raw sequence data have been deposited in the relevant International Nucleotide Sequence Database Collaboration (INSDC) databases under the Study ID PRJEB35151 (see Table S1 for run accessions).

**Table 1:** Voucher and repository information for the strains used in this study.

Species	Strain	Origin
*P. rubens*	IMI 15378	Alexander Fleming’s isolate from contaminated agar plate, London, UK^2^
*P. rubens*^a^	P2niaD18	Derived by mutagenesis and artificial selection from the IMI 015378 isolate, from mouldy cantaloupe, Illinois, USA^15^
*P. rubens*^a^	Wisconsin 54-1255	Derived by mutagenesis and artificial selection from the IMI 015378 isolate, from mouldy cantaloupe, Illinois, USA^15^
*P. nalgiovense*	IBT 13039	Goat cheese, Crete, Greece^56^

^a^Originally misnamed as *P. chrysogenum* but confirmed by molecular data to belong to the *P. rubens* clade^3,4^.

### Penicillin pathway genes

We searched for the *pcbAB*, *pcbC* and *penDE* genes in each genome using BLAST, and for a paralog of *penDE* that was first discovered in the Wisconsin 54-1255 genome^17,38^ and functionally characterized^39^. Query sequences are listed in Table S2. We also searched for *cefD1* and *cefD2* genes, which catalyse an alternative pathway for converting isopenicillin N to penicillin N rather than penicillin G, and were previously discovered in the Wisconsin 54-1255 genome^17^ and shown to be expressed. In addition, we searched for a suite of genes identified as playing a regulatory role in penicillin production: *anBH1*, the three subunits of the transcription factor *ancF* (*hapB*, *hapC*, and *hapE*), *pacC*, and *veA*. Functions of these genes are summarised in Fig. 1. In brief, PacC is a wide domain pH and carbon source dependent regulator*,* which upregulates the *pcbAB* and *pcbC* genes in *P. chrysogenum* in an alkaline environment and/or when the fungus is grown on a depleted carbon source^40,41^. VeA is a wide domain light dependent regulator in *P. chrysogenum*, *A. chrysogenum* and *Aspergillus nidulans*. It is involved in upregulation of *pcbAB* and downregulation of *pcbC*^26,42,43^. The transcription factor ancF consists of 3 subunits hapB, hapC and hapE and is responsible for the downregulation of the gene *pcbAB* and upregulation of the genes *pcbC* and *penDE* in *P. chrysogenum*^44–46^. The *anBH1* gene produces the basic-region helix loop helix protein (bHLH) that binds to the promoter region upstream of *penDE* and downregulates the transcription of *penDE*^26,47^.

For each gene, we generated an alignment (including multiple copies where present) using MAFFT 1.3.6^48^ and reconstructed a phylogenetic tree by maximum likelihood using the GTR + invgamma model in PHYML 2.2.3^49^, implemented in Geneious 9.1.8 (Biomatters Ltd, Auckland, New Zealand, https://www.geneious.com). We tested for evidence of positive selection among strains by running codon models in PAML 4^50^ for genes displaying variation: the null model of a single dN/dS ratio across codons, referred to as ω; the neutral model with a fraction *p*_1_ of codons that are under purifying selection (dN/dS < 1; *ω*_1_) and a fraction *p*_2_ evolving neutrally (dN/dS = 1; *ω*_2_); and the positive selection model including an additional fraction *p*_3_ codons evolving positively (dN/dS > 1; *ω*_3_). We used the Akaike Information Criterion to select the best model while penalizing for differences in the number of free parameters (null = 1, neutral = 2, positive = 4). The test is conservative because it requires substantial changes in amino acids to detect positive selection, whereas in reality a single amino acid change could underlie functional divergence. In addition to comparing best models, we plotted the average dN/dS ratio across the genome (ω) as a measure of degree of amino acid conservation among strains, with low values indicating stronger overall purifying selection.

## Results

### Genome assembly of *P. rubens* (IMI 15378)

A total of 2.86 Gb trimmed data (82.3% raw; Table S1) were used to generate an initial assembly comprising 274 scaffolds spanning 30.51 Mb. Screens for potential contamination resulted in the removal of 6 scaffolds (15.7 kb) that were not marked as from the genus *Penicillium* based on sequence similarity to NCBI ‘nt’ and UniRef90 public repositories (Fig S1). Scaffolds less than 500 bp in length were also discarded, resulting in a final draft assembly of 101 scaffolds spanning 30.46 Mb total length (~ 94X coverage), with an N50 scaffold length of 1.62 Mb. Assembly quality, based on the presence of core eukaryotic and fungal genes (BUSCO), indicated a high level of gene completeness (99.0% and 99.3% respectively) and a low level of duplication (0.3% and 1.0% respectively), suggesting a haploid genome assembly in common with the other strains (Table 2). The assembly is marginally smaller than the assemblies of the two industrial strains (32.4 and 32.2 Mb respectively) and has similar GC content (48.9% vs 49% for both industrial strains). The final genome assembly for *P. rubens* IMI 15378 (nPRUBv1) has been deposited at DDBJ/ENA/GenBank under the accession GCA_902636305.1 (CACPRF010000001–CACPRF010000101).

**Table 2:** Genome assembly metrics for *P. rubens* (IMI 15378) and the published genomes.

Strain	Fleming IMI 15378	P2niaD18^20^	Wisconsin 54-1255^17^	*P. nalgiovense* IBT 13039^56^
GenBank assembly accession (name)	GCA_902636305.1 (nPRUBv1)	GCA_000710275.1 (ASM71027v1)	GCA_000226395.1 (PenChr_Nov2007)	GCA_002072425.1 (ASM207242v1)
Coverage (method)	93X (Illumina HiSeq)	110X (Illumina HiSeq)	9.8X (clone-based Sanger)	115X (Illumina HiSeq)
Span (Mb)	30.5	32.5	32.2	33.0
No. scaffolds	101	5	49	1956
Scaffold N50 (kb)	1619	10,455	3889	358
Longest scaf fold (kb)	3393	13,598	5624	2018
% GC	49.0%	48.9%	49.0%	48.5%
BUSCO_EUK_ (*n* = 303)	C: 99.0% [S: 98.7%, D: 0.3%], F: 0.0%, M: 1.0%	C: 99.0% [S: 98.7%, D: 0.3%], F: 0.0%, M: 1.0%	C: 99.0% [S: 98.7%, D: 0.3%], F: 0.0%, M: 1.0%	C: 99.0% [S: 98.7%, D: 0.3%], F: 0.0%, M: 1.0%
BUSCO_FUN_ (*n* = 290)	C: 99.3% [S: 98.3%, D: 1.0%], F: 0.0%, M: 0.7%	C: 99.7% [S: 99.0%, D: 0.7%], F: 0.0%, M: 0.3%	C: 99.6% [S: 98.6%, D: 1.0%], F: 0.0%, M: 0.4%	C: 99.7% [S: 99.0%, D: 0.7%], F: 0.0%, M: 0.3%

BUSCO notation: C, complete BUSCOs; S, complete and single-copy BUSCOs, D, complete and duplicated BUSCOs; F, fragmented BUSCOs; M, missing BUSCOs.

### Structural comparison among Penicillium genomes

**Figure 2:**
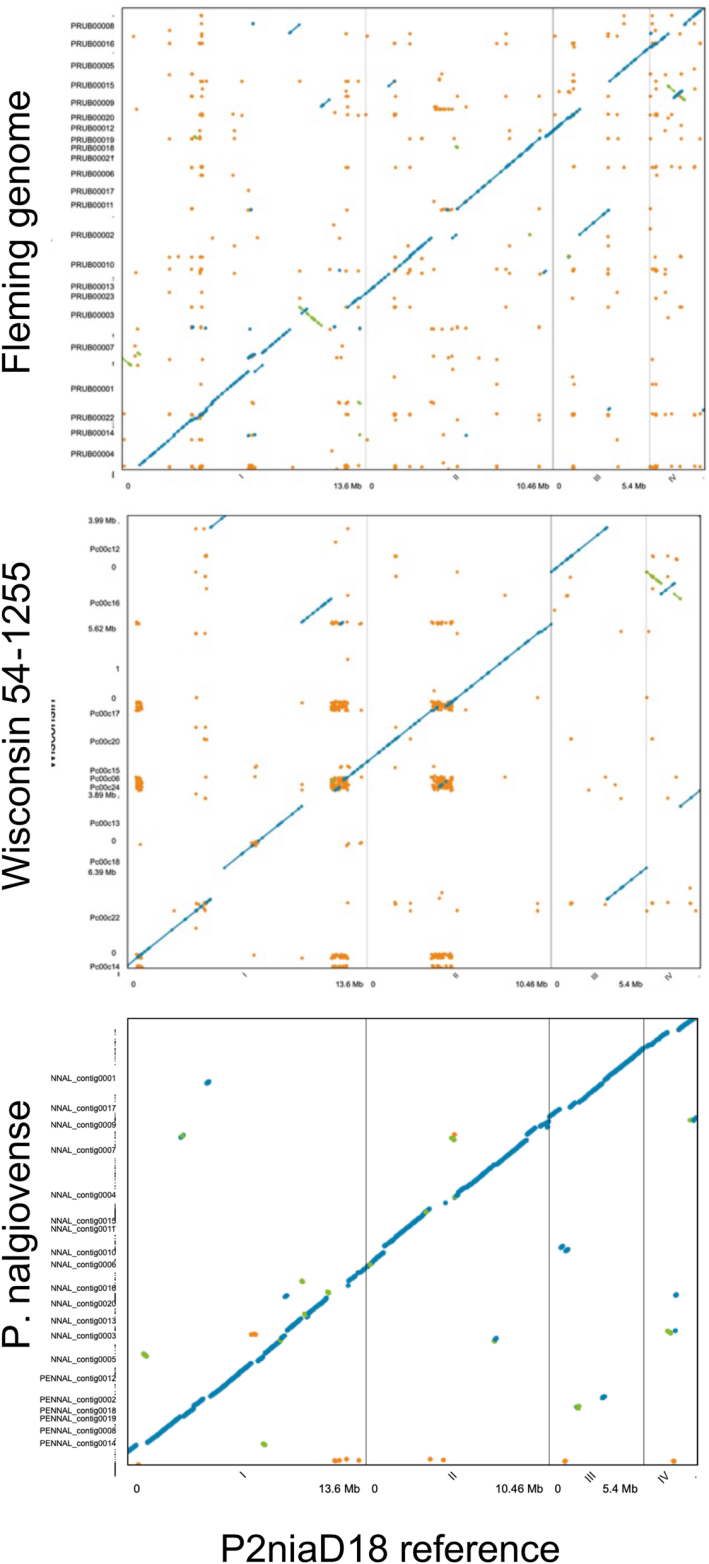
Dotplots showing regions of forward alignment (in blue), reversals (green) and repetitive alignments (orange). The 4 nuclear chromosomes of the ‘*P. chrysogenum*’ P2niaD18 assembly were used as the reference in each case: top panel, Fleming’s strain *P. rubens* (IMI 15378); middle panel, Wisconsin 54-1255; bottom panel, *P. nalgiovense* (IBT 13039).

The genome of Fleming’s *P. rubens* (IMI 15378) is broadly colinear with the P2niaD18 genome that was assembled to whole chromosome level, with relatively few cases of translocation or transversions (Fig. 2). More rearrangements are apparent between the Wisconsin 54-1255 strain and the P2niaD18 genome, perhaps indicative of structural mutations caused by mutagenesis during the improvement process as previously reported^20^. The *P. nalgiovense* IBT 13039 genome was broadly colinear with P2niaD18, although the greater fragmentation of the assembly makes it harder determine any large-scale rearrangements. All three genomes of *P. rubens* are highly similar at the sequence level: the Fleming genome is 0.106% divergent from the P2niaD18 genome across aligned regions (31,547 SNPs from 29.7 Mb alignment) whereas the Wisconsin 54-1255 is only 0.0038% divergent (1231 SNPs from 32.2 Mb alignment). *P. nalgiovense* IBT 13039 is 5.8% divergent (1,484,129 SNPs from 24.8 Mb aligned regions).

**Figure 3:**
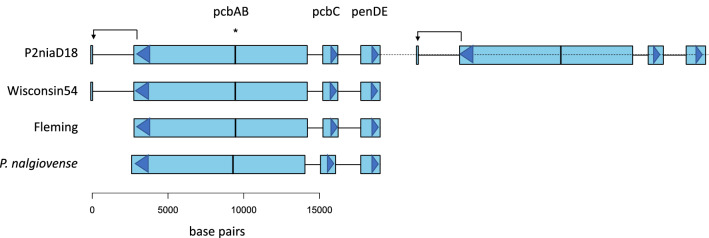
Structure of the penicillin gene cluster in the four strains. Distance between tandem duplicates in P2niaD18 not shown to scale (indicated by dashed line). The asterisk and vertical line in *pcbAB* indicate a fragment matching to a 36 bp fragment of the *cefD1* gene. More detailed view of the region is in figure S2.

The structure of the penicillin effector genes is conserved across species and always falls into the well characterized cluster of *pcbAB*, *pcbC* and *penDE* genes (Fig. 3). The P2niaD18 genome alone has a single tandem duplication of the whole cluster, rather than multiple complete or partial tandem duplications of the cluster present in other industrial penicillium strains^20,51,52^. In addition to the main loci, a partial duplicate exhibiting a match to the final 123 bp of *pcbAB* but with three amino acid substitutions is found in a non-coding region in the two industrial strains, 2704 bp downstream of *pcbAB* (Fig. 3, Table S3). This fragment, labelled B1 in the previous analysis of Wisconsin 54-1255 by Fierro et al*.*^52^ is found in both tandem duplicates in P2niaD18, but absent from the Fleming genome (as confirmed by mapping raw reads of Fleming strain onto the Wisconsin 54-1255 genome, Fig. S2). We speculate that this might play a functional role in the region, for example in regulating expression of *pcbAB*, but it might simply be a neutral or deleterious side-effect of the mutagenesis during improvement of those strains.

Other putative beta-lactam effector genes were found in the genomes of all the four strains compared. All four strains contained the paralog of *penDE* first identified in the Wisconsin 54-1255 genome^17^. The *cefD1* region contains additional 36 to 57 bp long fragments that blast to genome regions outside the main coding region. BLAST confirmed that these are not repeated domains or regions found elsewhere in the genome but represent single partial duplications similar to those observed in *pcbAB*. Two of these duplicate fragments were found only in the three *P. rubens* strains and two only in *P. nalgiovense*. The *cefD2* region was not duplicated but recovered as two long sections and one short section in all three genomes, indicating the absence of match across the full-length region found in the *P. arizonense* gene used for the query.

The genes involved in regulation of penicillin production were scattered across the genome of each strain (Table S3). The *hapB* gene in the *ancF* transcription factor complex displays a partial duplicated fragment of 95 bp in the two industrial strains, which is lacking in the other two genomes. A clear *hapE* match was missing for *P. nalgiovense*. All other regulatory genes are present in single copy in all four genomes.

### Sequence divergence of penicillin effector and regulatory genes

**Figure 4:**
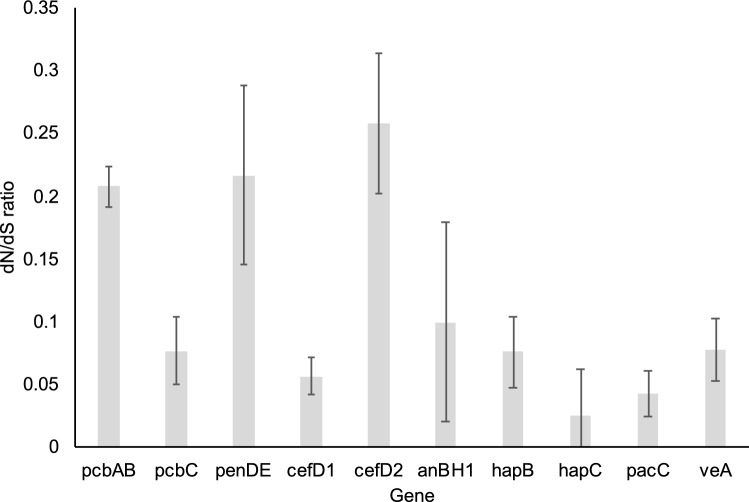
The average ratio of non-synonymous to synonymous substitutions (dN/dS) for alignments of penicillin pathway genes across the sampled genomes: standard error bars on the estimate from the PAML analysis are shown. The first four genes were sampled for three strains: Wisconsin 54-1255, Fleming (IMI 15378), and *P. nalgiovense* (IBT 13039). The remaining genes were compared just between the Fleming (IMI 15378) genome and *P. nalgiovense* (IBT 13039) because of the lack of any variation across the three *P. rubens* strains.

The two industrial strains, P2niaD18 and Wisconsin 54-1255, were identical at the sequence level for all the focal genes and therefore for subsequent analyses only sequences from Wisconsin 54-1255 were used to represent the American isolate of *P. rubens*. In contrast, penicillin-pathway genes have diverged in amino acid sequence between the Fleming strain and the US strains. All three effector genes encoding enzymes in the penicillin G pathway have diverged, but *pcbAB* and *penDE* showed the highest rates of amino acid divergence relative to silent changes whereas *pcbC* was strongly conserved (Fig. 4, Table S4). The level of divergence in *pcbAB* is unexpected since this gene functions to produce the initial precursor in the pathway, which is shared in the production of other beta-lactams. The homologs of *cefD1* and *cefD2* genes were found in all genomes of *P. rubens* strains. This was unexpected as these genes are involved in the synthesis of the cephalosporin intermediate penicillin N in *A. chrysogenum* and are not known to have a functional role in *P. rubens*^17,53,54^. The penicillin N effector gene *cefD2* also showed a high level of amino acid divergence whereas *cefD1* was more strongly conserved than other effector genes. The best sequence model for the effector genes plus *hapB* was a model with most codons being under constraint (dN/dS < < 1) but with a significant proportion of codons being unconstrained (dN/dS = 1). There was no sequence divergence between American and British *P. rubens* isolates in the *penDE* paralog, *pacC*, *ancF* (*hapB*, *C* and *E*) or *veA*. To further investigate possible regulatory changes, we looked for sequence variation within transcription factor binding sites within the intergenic region between *pcbAB* and *pcbC*, which is a bidirectional promotor region for these genes. Among 28 binding sites previously identified in *P. chrysogenum*^55^, all were found in the Fleming genome, and just one site was lost in both industrial strains (GATA to GGTA mutation, Table S5). Thus, the divergence of known binding sites is low, similar to that seen for regulatory proteins.

## Discussion

Nearly a century since Alexander Fleming discovered the action of penicillin in bacterial cultures contaminated by *P. rubens*, we report the first draft genome sequence of his original strain. Very soon after the original discovery and isolation of penicillin, a second wild isolate of *P. rubens* from the USA was employed for future industrial manufacture owing to its greater rate of penicillin production^15^. Consequently, two of the strains derived from this isolate have been the focus of previous whole genome sequencing within the *P. rubens* clade^16,17,20^. We compared these genomes with each other and a third, more distantly related genome of *P. nalgiovense*^56^.

Comparison of the two USA strains provides insights into the industrial mutagenesis and artificial selection process^16^, which was originally performed by selecting phenotypically useful mutants without knowledge of the underlying genomic basis^15^. There were no amino acid differences at any genes encoding the enzymes in the penicillin pathway and regulatory genes. Instead, there was evidence for structural rearrangement across the genome, including tandem duplication of the *pcbAB*–*pcbC*–*penDE* cluster in P2niaD18, which has previously been studied in these and other industrial strains^51,52^. This fits with the type of mutagenesis and artificial selection used for this process. Experimental work showed that tandem duplication of the *pcbAB*–*pcbC*–*penDE* cluster does not directly increase penicillin production over short time periods—a strain of P2niaD18 that was modified to lose one copy did not produce significantly less penicillin over a 96-h assay period^57^. Substantial copy number multiplication of the region among industrial strains still seems to implicate gene duplication in penicillin production, but perhaps only under specific growth conditions or over longer periods^18,51^. Another plausible source of variation would be changes in regulatory regions, but experimental evidence indicates that such variation is unlikely to contribute to increased penicillin production^18^.

Comparison between the UK and US genomes sheds light on both evolved differences between the wild progenitors of the strains, and potential initial changes in the domestication steps prior to the divergence of P2niaD18 and Wisconsin 54-1255. One structural difference shared by the US genomes was the partial duplication of the final portion of the *pcbAB* gene. Read mapping confirmed that this region is missing from the Fleming genome and not just absent due to assembly artefacts (Fig. S2). Partial duplication and inversion have been documented previously at the ends of the amplified region containing the penicillin synthesis genes for Wisconsin 54-1255^52^. Furthermore, partial duplication has been found to play a role in generating novel diversity previously, e.g., in the case of pathogen resistance in barley^58^, and could play a role in gene regulation. Without further sequencing, we cannot be certain whether this change occurred in the wild progenitor of the US strains or during initial stages of domestication. Because of the nature of these changes in relation to the predicted effects of mutagenesis, however, and the fact that further such differences arose between P2niaD18 and Wisconsin 54-1255, it seems plausible that shared structural differences of the two industrial strains from the Fleming genome occurred during their initial shared history of mutagenesis prior to their separation. No sequence divergence was observed between the two US strains in any of the genes involved in penicillin G production and regulation of the pathway: mutagenesis and selection for improved function resulted in major structural changes but no substitutions at these loci.

In contrast, penicillin-pathway enzymes have diverged in amino acid sequence between the Fleming strain and the US strains, especially *pcbAB*, *penDE* and *cefD2*. While it is possible in principle that these changes were caused by mutagenesis during domestication of the US strains, we think that this is unlikely: subsequent rounds of the same process led to no sequence divergence between the US strains, and the numbers of substitutions involved would seem more commensurate with longer periods of time elapsing. Instead, these differences are likely to have accrued during evolutionary divergence of the UK and US strains of *P. rubens* in the wild.

Although the level of divergence did not meet the statistical criteria for detecting significant evidence of positive selection, a low level of constraint on protein sequence of these genes could still indicate a history of divergent selection at a subset of codons. Alternatively, it could indicate that the function of these proteins is less dependent on amino acid identity at several sites than is the case for the other genes. In *A. nidulans*, the *aatA* gene (an ortholog of *penDE*) encodes the enzyme isopenicillin N acyltransferase^26,59^. It has been found that disruption of this gene does not disrupt penicillin production in *A. nidulans*. A paralog of *aatA*, *aatB* compensates for this as it encodes a homolog of isopenicillin N acyltransferase^59^. It should be noted that the isopenicillin N acyltransferase encoded by *aatA* is only 55.2% similar to its homolog encoded by *aatB* and the two genes themselves are only 58% similar^59^.

Additionally, the liquid chromatography–mass spectrometry (LC–MS) data for penicillin compounds synthesized by either of the genes indicate unexplained significant peaks in proximity to the peaks representing standard penicillin V or penicillin G compounds synthesized by these genes. These unexplained peaks could represent penicillin analogues synthesized by *aatB* and *aatA*. It would be worthwhile to investigate further how the differences in penicillin effector genes translate into altered function of the enzymes encoded, such as variation in the substrate specificity or efficiency of the enzymes^60^. Such variation in specificity of the enzymes could result in synthesis of penicillin G analogues. Furthermore, presence of a *penDE* paralog, and *cefD1* and *cefD2* homologs in all the genomes compared in this study suggest the possibility that these genes encode homologs of isopenicillin N acyltransferase and isopenicillin N epimerase respectively^17,39,53,61^. These enzymes could potentially synthesize analogues of penicillin G and penicillin N. Other beta-lactam gene variants such as homologs of the gene encoding 7-alpha-cephemmethoxylase subunit, *cmcJ*, have also been identified in the genome of *P. chysogenum*^17^. Studies suggest that many of these gene variants are expressed but further work is needed to elucidate the functional importance of these genes, which is currently unclear^17,39,54^.

The biosynthesis of penicillin G in *P. chrysogenum* and *P. rubens* consists of a simple three gene pathway, but in certain bacteria such as *S. clavuligerus*, as many as twelve genes can be involved in the synthesis of beta-lactams such as cephamycin C^26,62^. Much of what is known regarding the evolution of diversity of natural antibiotics stems from the concept of rearrangement of genes in an existing biosynthetic gene cluster, or by addition of novel genes to existing clusters via processes such as horizontal gene transfer^12,63^. Our analyses indicate that individual genes of beta-lactam biosynthetic pathways can themselves vary between species. Evidence indicates that many penicillin producing species such as *P. chrysogenum* are genetically diverse, and allelic variation within wild *P. chrysogenum* populations can impact penicillin production within these populations^64,65^. Thus, it is plausible that sequence variation in the genomes that we describe could account for the production of novel penicillin analogues. Subtle variation in chemical structure of antibiotics has been identified for other antibiotics such as antimycins produced by *Streptomyces*^63,66,67^. Future work to sample variation more widely in *P. rubens* and measure the impacts of variation on chemical structure of penicillin compounds is needed to distinguish these alternatives.

In conclusion, our results provide preliminary evidence that genes involved in the production of penicillin display relatively high rates of amino acid divergence between populations, as predicted if antibiotics evolve in an arms race with antagonistic microbes. Moreover, the results indicate that natural changes involving point mutation and amino acid substitutions were not fully explored by the classical industrial mutagenesis approach, which instead produced larger structural rearrangements. Thus, the mutagenesis approach employed previously may have missed some solutions for optimizing penicillin design compared to natural selection in the wild, especially in the context of robustness to evolving antibiotic resistance. Future approaches could use solutions explored by nature as a template for the development of novel antibiotic varieties.

## Supplementary information

Supplementary Information.
