# Supplementary material for: Comparative genomics of Alexander Fleming’s original Penicillium isolate (IMI 15378) reveals sequence divergence of penicillin synthesis genes
Source: Sci Rep. 2020 Sep 24;10:15705. doi: 10.1038/s41598-020-72584-5 (PMC7515868; doi:10.1038/s41598-020-72584-5)
Supplement: Supplementary file 1 — Supplementary Information. [file 41598_2020_72584_MOESM1_ESM.pdf]

SUPPLEMENTARY INFORMATION

Comparative genomics of Alexander Fleming's original *Penicillium* isolate (IMI 15378) sheds light on the evolution of penicillin synthesis genes

Ayush Pathak[1], Reuben W. Nowell[1,2], Christopher G. Wilson[1,2], Matthew J. Ryan[3] and Timothy G. Barraclough[1,2,*]

[1]Department of Life Sciences, Imperial College London, Silwood Park Campus, Ascot, Berkshire SL5 7PY, UK
[2]Department of Zoology, University of Oxford, 11a Mansfield Rd, Oxford OX1 3SZ, UK.
[3]CABI, Bakeham Lane, Egham, Surrey TW20 9TY UK
*Corresponding author: tim.barraclough@zoo.ox.ac.uk

Table S1: Data counts for P. rubens IMI 15378

**Library name**	**Library type**	**Insert size (bp)**	**Number of paired reads (raw)**	**Number of paired reads (trimmed)**	**Number of bases (trimmed)**	**SRA accession**
Anc_S4	150 bp PE	400	3,969,072	3,966,144	596,228,858	ERR3630060
EFEB_S1	150 bp PE	400	4,575,872	4,569,964	68,680,634	ERR3630061
EFEB-NDIZ_S3	150 bp PE	400	3,001,622	2,998,460	450,707,400	ERR3630062
EFSB_S2	150 bp PE	400	3,680,926	3,676,174	552,455,559	ERR3630063
SF5_S5	150 bp PE	400	5,210,698	5,204,128	782,105,378	ERR3630064
SF6_S6	150 bp PE	400	2,734,910	2,731,536	410,521,026	ERR3630065
**TOTAL**			**23,173,100**	**23,146,406 (99.9%)**	**2,860,698,855 (82.3%)**	

Table S2. Query sequences used to BLAST penicillin pathway genes

Gene	Organism	Genbank accession numbers
pcbAB	*P. rubens* Wisconsin 54-1255	NW_003020110
pcbC	*P. rubens* Wisconsin 54-1255	NW_003020110

penDE	*P. rubens* Wisconsin 54-1255	NW_003020110
penDE paralog	*P. 'chrysogenum'* P2niaD18	XM_002559293
pacC	*P. rubens* Wisconsin 54-1255	NW_003020078
cefD1	*P. arizonense* CBS 141311	NW_019173322
cefD2	*P. arizonense* CBS 141311	NW_019173279
hapB	*P. digitatum* Pd1	NW_014574624
hapC	*P. digitatum* Pd1	NW_014574583
hapE	*P. digitatum* Pd1	NW_014574615
veA	*P. digitatum* Pd1	NW_014574610
anBH1	*Aspergillus nidulans FGSC A4*	NT_107006

Table S3. Penicillin pathway genes recovered by BLAST from the 3 genomes

	P. 'chrysogenum' P2niaD18			*P. rubens* Wisconsin 54-1255			*P. rubens* Fleming IMI 15378			*P. nalgiovense* IBT 13039		
Gene	chr	position	length	contig	position	length	contig	position	length	contig	position	length
pcbAB	II	9756972 - 9768342	11371	1	5034016 - 5045386	11371	PRUB00025	31142 - 42515	11374	0007	731682 - 720312	11371
	II	9771046 - 9771168	123	1	5048090 - 5048212	123						
	II	9862193 - 9873563	11371									
	II	9876267 - 9876389	123									
pcbC	II	9861177 - 9860182	996	1	5033000 - 5032005	996	PRUB00025	30125 - 29130	996	0007	732700 - 733691	992
	II	9755956 - 9754961	996									
penDE	II	9858683 - 9857409	1275	1	5030506 - 5029232	1275	PRUB00025	27646 - 26372	1275	0007	735341 - 736616	1276
	II	9753462 - 9752188	1275									
penDE paralog	I	9332981 - 9334190	1210	Pc00c13	2228128 - 2229337	1210	PRUB00007	1613315 - 1614524	1210	0003	485443-486117	675

*cefD1**	II	527561 - 525553	2009	Pc00c20	3127137 - 3129145	2009	PRUB00013	201635 - 199627	2009	0006	148113- 146106	2008
	I	13501597 - 13501542	56	Pc00c15	93858 - 93913	56	PRUB00003	95883 - 95938	56			
	II	9761673 - 9761708	36	1	5038717 - 5038752	36	PRUB00025	35843 - 35878	36	0007	726981- 726946	36
	II	9866894 - 9866929	36									
	I	6854451 - 6854485	57	Pc00c18	1357407 - 1357351	57	PRUB00001	1074200 - 1074256	57			
										0012	37695- 37756	62
										0010	222194- 222168	27
*cefD2**	I	3705426 - 3706548	1123	Pc00c22	3237038 - 3235916	1123	PRUB00014	111924 - 110802	1123	0019	214097- 215219	1123
	II	5002487 - 5003578	1092	1	278237 - 279328	1092	PRUB00002	912398 - 913489	1092	0007	305748- 306855	1108
	II	5001865 - 5001898	34	1	277615 - 277648	34	PRUB00002	911776 - 911809	34	0007	305159- 305192	34
hapB†	III	373681 - 374765	1085	Pc00c12	371686 - 372770	1085	PRUB00012	387180 - 388264	1085	0009	615788- 616873	1086
	I	12352993 - 12353087	95	Pc00c24	83319 - 83225	95						
hapC†	I	377843 - 378481	639	Pc00c14	376058 - 376696	639	PRUB00001	372933 - 373571	639	0038	203023- 202385	639
hapE†	III	1052417 - 1053534	1118	Pc00c12	1050500 - 1051617	1118	PRUB00012	984822 - 985939	1118	0006	843619- 844732	1114
pacC	I	5601033 - 5603020	1988	Pc00c18	103869 - 105856	1988	PRUB00001	2324907 - 2322920	1988	0002	101220- 99225	1996
veA	IV	2352979 - 2354713	1718	Pc00c13	3199729 - 3201463	1718	PRUB00008	607842 - 606108	1718	0031	238341- 236605	1737
anBH1	I	3190618- 3190166	453	Pc00c22	3751951- 3752403	453	PRUB00014	628087- 628539	453	0057	22454- 22002	453

* The short fragments (30-60bp) only single blast to *P. arizonae*, so not repeat element or common protein motif
† Subunits of the *ancF* complex transcription factor

P2niaD18 has tandem duplication of *pcbAB*, *C*, *penDE* region

Table S4. **Sequence evolution among the genome strains inferred by fitting codon evolution models in PAML**. The first four genes showed variation between the US and British isolates of *P. rubens* and models were run on alignments with 3 sequences: Wisconsin 54-1255, Fleming strain and *P. nalgiovense*. The remainder showed no variation within *P. rubens* (i.e. Fleming, P2niaD18 and Wisconsin 54-1255) and models were run on an alignment with just the Fleming strain and *P. nalgiovense*. Bold indicates the preferred model with lowest Akaike Information Criterion (AIC) for each gene.

Gene	% div within *P. rubens*		% div to *P. nalgiovense*		Model	Model parameters						AIC
	silent	coding	silent	coding		*w1*	*p1*	*w2*	*p2*	*w3*	*p3*	
pcbAB	3.01	0.64	13.2	2.73	0)	0.208	1.0					37493.98
					**1)**	**0.141**	**0.915**	**1.0**	**0.085**			**37488.75**
					2)	0.162	0.949	1.0	0.047	3.614	0.0039	37492.47
pcbC	6.89	0.53	14.6	1.02	0)	0.077	1.0					3024.29
					**1)**	**0.000**	**0.914**	**1.0**	**0.086**			**3016.81**
					2)	0.000	0.038	1.0	0	1.761	0.062	3020.17
penDE	2.61	0.57	8.10	1.76	0)	0.217	1.0					3345.12
					**1)**	**0.000**	**0.789**	**1.0**	**0.211**			**3341.35**
					2)	0.068	0.936	1.0	0	3.032	0.064	3344.43
cefD1	0	0	20.1	1.14	0)	0.057	1.0					5925.26
					**1)**	**0.000**	**0.937**	**1.0**	**0.063**			**5918.47**
					2)	0.023	0.982	1.0	0	2.720	0.018	5922.30
cefD2	0.42	0.11	15.1	3.90	0)	0.258	1.0					4963.86
					**1)**	**0.022**	**0.735**	**1.0**	**0.265**			**4961.87**

					2)	0.230	0.993	1.0	0	18.452	0.007	4962.34
anBH1	0	0	10.3	1.02	**0)**	**0.100**	**1.0**					**1152.5**
					1)	0.100	1.0	1.0	0			1154.5
					2)	0.100	1.0	1.0	0	1.0	0	1158.5
*hapB**	0	0	14.3	1.09	0)	0.076	1.0					3285.08
					**1)**	**0.000**	**0.922**	**1.0**	**0.078**			**3282.57**
					2)	0.052	0.995	1.0	0	23.112	0.005	3284.61
hapC	0	0	8.45	0.21	**0)**	**0.025**	**1.0**					**1815.50**
					1)	0.025	1.000	1.0	0.000			1817.50
					2)	0.025	1.000	1.0	0.000	1.000	0.000	1821.50
pacC	0	0	12.0	0.52	**0)**	**0.043**	**1.0**					**5644.22**
					1)	0.043	1.000	1.0	0.000			5646.22
					2)	0.043	1.000	1.0	0.000	1.000	0.000	5650.22
veA	0	0	13.6	1.06	**0)**	**0.078**	**1.0**					**5114.59**
					1)	0.078	1.000	1.0	0.000			5116.59
					2)	0.078	1.000	1.0	0.000	1.000	0.000	5120.59

**hapE* not shown as all sequences identical in all three compared strains

Table S5 Sequence variation within the intergenic pcbAB-pcbC bidirectional promotor region among the *P. rubens* strains.

Binding motif	Associated with regulatory gene	Number of motifs within promotor region	Variation affecting binding sites
TATA		3	none
CCAAT	ancF/hap	6	none
GCCARG	pacC	7	none
SYGGRG	creA	6	none
GATA	areA (NRE)	6: Fleming IMI 15378<br>5: P2niaD18 and Wisconsin 54-1255	One GATA mutated to GGTA in industrial strains

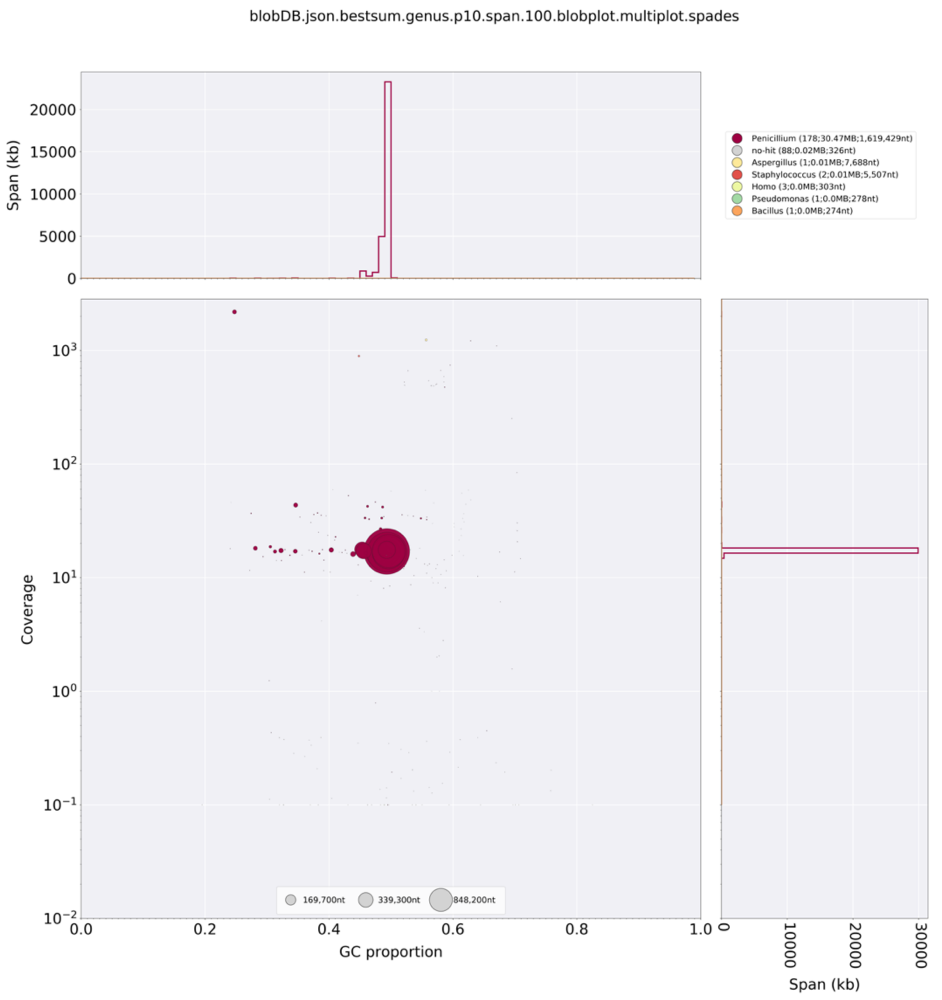

Figure S1: Taxon-annotated GC–coverage plot ("blobplot") for 274 scaffolds generated during initial assembly. Circles represent assembled scaffolds plotted based on %GC (*X*-axis) and coverage (*Y*-axis), coloured by taxon (based on sequence similarity, shown at the genus level). The lot indicates negligible presence of non-target sequences in the assembly.

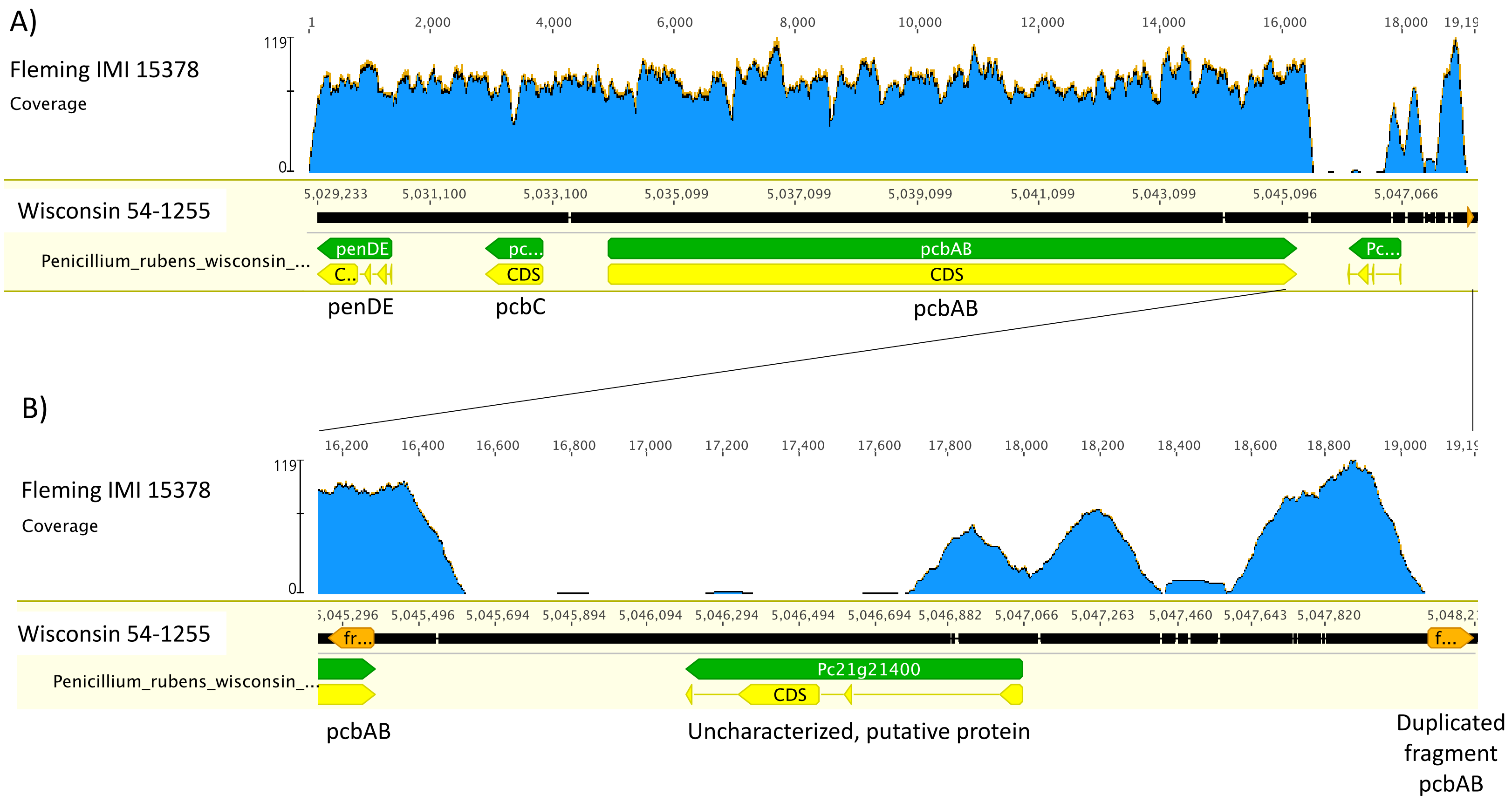

Figure S2. The pcbAB-penDE region of the Wisconsin 54-1255 genome showing the coverage when reads from the Fleming IMI 15378 genome were mapped onto it. (A) Full region. (B) Zoomed in view of the upstream region of pcbAB showing the location of an uncharacterized predicted protein and of the duplicated 123bp fragment of the terminal end of the pcbAB gene. Coverage plot demonstrates that both the gene and the duplicated fragment are absent in the Fleming genome and not just missing from the assembled genome due to assembly artefacts.
